# Simulated Microgravity Altered the Metabolism of Loureirin B and the Expression of Major Cytochrome P450 in Liver of Rats

**DOI:** 10.3389/fphar.2018.01130

**Published:** 2018-10-12

**Authors:** Bo Chen, Jingjing Guo, Shibo Wang, Liting Kang, Yulin Deng, Yujuan Li

**Affiliations:** School of Life Science, Beijing Institute of Technology, Beijing, China

**Keywords:** simulated microgravity, dragon blood, loureirin B, liver microsome, hepatic cytochrome P450, drug metabolism

## Abstract

Loureirin B (LB) is the marker compound of dragon blood (DB), which exhibits great potentials in protecting astronauts’ health against radiation and simulated microgravity (SM). Pharmacokinetics of LB is reported to be significantly altered by SM. Here, we investigated key metabolic features of LB in rat liver microsome (RLM) and the effects of SM on LB metabolism as well as on expression of major hepatic cytochrome P450 (CYP450) isoforms. Ten metabolites were tentatively identified based on fragmentation pathways using LC-MS/MS method and elimination kinetics of LB followed a typical Michaelis–Menten equation (*V*_*max*_ was 1.32 μg/min/mg and *K*_*m*_ was 13.33 μg/mL). CYP1A2, CYP2C11, CYP2D1, and CYP3A2 were involved in the metabolism of LB and the relative strength was: CYP3A2 > CYP2C11 > CYP2D1 > CYP1A2. Comparative studies suggested that elimination of LB in RLM was remarkably increased by 3-day and 14-day SM, and the generation of identified metabolites was affected as well. Additionally, 3-day and 14-day SM showed obvious regulatory effects on the expression of major CYP450 isoforms, which might contribute to the increased elimination of LB. The data provided supports for the application of DB as a protective agent and the reasonable use of current medications metabolized by hepatic CYP450 in space missions.

## Introduction

**FIGURE 1:**
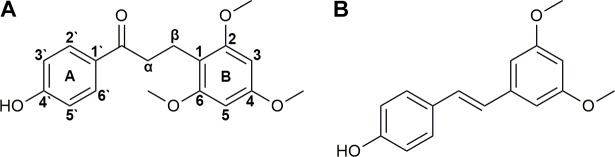
Chemical structures of loureirin B **(A)** and pterostilbene **(B)**.

Since the beginning of manned spaceflight in 1967, medications have been introduced into missions (Graebe et al., 2004) for complaints induced by adverse environmental factors, such as intense radiation, high vacuum, extreme temperature, disrupted circadian rhythms, and microgravity (Setlow, 1999) in the space. Traditional Chinese medicine is recently appreciated for its potentials in protecting astronauts’ cardiovascular, skeletomuscular and endocrine systems (Fan et al., 2003, 2004; Li et al., 2003, 2016; Zhang et al., 2005; Li, 2008; Zhang and Xie, 2008, 2009; Zhou et al., 2008; Sun, 2012; Zhu et al., 2012). DB is the red resin produced by *Dracaena cochinchinensis* and rich of bio-active components (Fan et al., 2014; Hao et al., 2015). It is historically used for the treatments of traumatic injuries, blood stasis and pains (Fan et al., 2014). Previous studies of our group demonstrated its potentials in the protection of astronauts against damage induced by radiation and SM (Xin et al., 2012; Li et al., 2013; Ran et al., 2014a,b, 2016; Chen et al., 2016). LB (**Figure 1A**) is the marker compound and commonly used for QC of DB (Chen et al., 2017). Many pharmacological tests have been performed suggesting that LB possesses various activities, such as improving blood circulation (Deng et al., 2004) and pain relief (Liu et al., 2004, 2005; Zhang et al., 2006; Wang et al., 2007; Yang et al., 2009).

Medications are applied in the space under the assumption that they function in a similar way as on the ground, which has not been fully verified yet (Kast et al., 2017). Up to now, several studies have suggested that microgravity or SM dramatically alters pharmacokinetic processes of medications (Derendorf, 1994; Tietze and Putcha, 1994; Graebe et al., 2004; Gandia et al., 2005; Kast et al., 2017). Pharmacokinetics of LB has been tested by our group and the results showed that it was also obviously altered by SM (Li et al., 2014; Chen et al., 2017). However, the mechanism of variations remains unclear.

Metabolism plays a key role in pharmacokinetics of medications. It facilitates the excretion of medications by increasing their hydrophilia. Liver acts as the dominant organ of metabolism due to its high blood perfusion and large contents of CYP450, a superfamily of heme-containing enzymes responsible for the metabolism of most endogenous compounds and xenobiotics including medications, pesticides, and pollutants (Tang et al., 2006; Vuppugalla et al., 2007). A previous study suggested that total CYP450 level in rat liver was changed when exposed to spaceflight (Merrill et al., 1990). Therefore, it is significant to investigate the metabolic features of LB in liver and especially the effects of SM on LB metabolism for understanding the pharmacokinetic variations.

CYP1A, CYP2C, CYP2D, CYP2E, and CYP3A predominate human drug metabolism (Tang et al., 2006; Qiu et al., 2008; Guo et al., 2014; Sun et al., 2014). Medications used in space missions such as promethazine, zolpidem, and ibuprofen are also metabolized by the five major isoforms (Hesse et al., 2003; Litzenburger et al., 2015; Ikuta et al., 2017). Up or down-regulation of the isoforms may significantly alter the metabolism of the up-mentioned medications leading to either failure of treatment or unexpected toxicity (Ren et al., 2008; Sun et al., 2014). However, the effects of microgravity on the expression of major CYP450 isoforms have not been studied systematically yet.

The present study was designed to investigate the metabolic features of LB (metabolites, elimination kinetics, and metabolizing CYP450) in RLM and the effects of different SM durations (3, 7, 14, and 21 days) on the metabolism of LB (elimination amounts of LB and relative generation amounts of metabolites) in RLM using rat tail-suspension model. Meanwhile, mRNA and protein levels of five major CYP450 (CYP1A2, CYP2C11, CYP2D1, CYP2E1, and CYP3A2) were investigated as well. The data might help to understand the pharmacokinetic alterations of LB in SM and thus support the application of DB as a protective agent for astronauts. Moreover, results of gene expression of major CYP450 might be of great importance for reasonable use of current medications in space missions.

## Materials and Methods

### Reagents and Drugs

Trizol and HPLC-grade acetonitrile were purchased from Thermo Fisher Scientific (Waltham, MA, United States). LB was supplied by National Institute for the Control of Pharmaceutical and Biological Products (Beijing, China). Pterostilbene (used as the IS, **Figure 1B**) was purchased from Great Forest Biomedical Ltd. (Hangzhou, China). NADPH-regenerating solution was obtained from iPhase Pharmaceutical Services (Beijing, China, containing 21.7 mM of NADP^+^, 55 mM of G-6-P, 6.67 U/mL of G-6-PDH and 55 mM of MgCl_2_). Cimetidine, α-naphthoflavone, quinindium, 4-methylpyrazole, and ketoconazole were from J&K Chemical (Beijing, China). Oligo(dT) 15 primer, RNase-free water, RNasin Plus RNase Inhibitor, 5× M-MLV Reverse Transcriptase buffer, M-MLV Reverse Transcriptase and dNTPs were obtained from Promega Corporation (Madison, WI, United States). SYBR^®^ Premix Ex Taq™ (Tli RNaseH Plus) was from TAKARA BIO INC. (Dalian, China). Enhanced RIPA lysis buffer and protein loading buffer were purchased from Solarbio Life Sciences (Beijing, China). Primary antibodies of CYP1A2, CYP2C11, CYP2D1, CYP2E1, and CYP3A2 were supplied by Abcam PLC (Cambridge, United Kingdom). Primary antibody of GAPDH was from Beyotime Biotechnology (Shanghai, China) and HRP-conjugated secondary antibody was from ComWin Biotech Co., Ltd. (Beijing, China). All other chemicals and solvents were of analytical grade.

### Animals and Liver Collection

The study complied with the Guide for the Care and Use of Laboratory Animals published by the National Institutes of Health (NIH publication no. 85-23, revised in 1985), and all animals were approved by Beijing Institute of Technology Animal Care and Use Committee. Forty-eight Sprague-Dawley rats (male, SPF, 200 ± 20 g) in total were purchased from Academy of Military Medical Sciences (Beijing, China). Rats were kept in an animal room with free access to water and food for 1 week prior to the study. Temperature, humidity and illumination were artificially controlled as follows: temperature 24°C, humidity 55%, illumination 12-h light-dark cycle.

The rats were randomly divided into eight groups (six rats per group): four control groups, rats were freely raised in normal cages for 3, 7, 14, and 21 days; four model groups, rats were tail-suspended (Morey-Holton and Globus, 2002) for 3, 7, 14, and 21 days to mimic different durations of SM. When the SM course ended, rats were fasted overnight and sacrificed by cervical dislocation. After the abdomen was opened, ice-cold 10 mM PBS buffer (pH 7.4) was injected into the liver portal vein and meanwhile postcava was cut open to release the perfusate. Once turned khaki, the liver was harvested and divided into two parts. One was stored at -80°C for RNA and protein extraction, and the other was immediately used for the preparation of microsome.

### Preparation of Liver Microsome

Liver microsome was prepared according to a validated method with a little modification (Tang et al., 2006). Briefly, 2 g of liver tissue was placed in a glass homogenizer, minced and homogenized in 6 mL of ice-cold 10 mM PBS buffer (pH 7.4). The homogenate was centrifuged at 10,000 ×*g* for 30 min at 4°C to remove cell debris, nuclei, and mitochondria. The supernatant was then centrifuged at 100,000 × *g* for 60 min at 4°C. The pellet was resuspended with fresh ice-cold 10 mM PBS buffer (pH 7.4) and centrifuged again at 100,000 ×*g* for 60 min at 4°C. The final pellet was resuspended with 20% glycerol-PBS and stored at -80°C until use.

### HPLC-UV Method Development for LB Quantification in RLM

Liver microsome from each individual rat was pooled equally for the development of the quantitative method.

#### Preparation of Calibration Curve and Quality Control Samples

Stock solutions of 10 mg/mL of LB and IS were prepared in acetonitrile. Calibration standard solutions of 0.1, 0.2, 0.5, 1, 2, 4, 8, and 10 mg/mL of LB were prepared by diluting stock solution in acetonitrile. Calibration curve samples were prepared by mixing 2 μL of calibration standard solutions with 176 μL of PBS, 10 μL of heat-denatured microsome (final protein concentration was 1 mg/mL), and 12 μL of NADPH-regenerating solution to give the final concentration of LB from 1 to 100 μg/mL. QC samples were prepared similarly at three final concentrations of 2.0 (low), 20 (medium), and 80 (high) μg/mL of LB. IS working solution was prepared by diluting stock solution in acetonitrile to give the final concentration of 10 μg/mL of IS. All solutions were stored at 4°C and avoid from light.

#### Sample Preparation for HPLC-UV Analysis

Four hundred microliters of ice-cold acetonitrile containing IS was added to samples to precipitate proteins. Precipitated samples were vortexed vigorously for 3 min at room temperature and then centrifuged at 12,000 ×*g* for 10 min at 4°C. The supernatant was used for HPLC-UV analysis.

#### Chromatographic Conditions

Loureirin B and IS were separated on a Rigol L-3000 HPLC system with an UV detector (RIGOL, China). Twenty microliters of the supernatant was injected into an Xtimate C_18_ column (4.6 mm × 150 mm, 5-μm particle, Welch, United States). Flow rate was set at 1.0 mL/min and column temperature was 30°C. Mobile phase was acetonitrile/water at the ratio of 60/40. Detecting wavelength was 280 nm.

#### Method Validation

##### Specificity

Chromatograms of blank microsome, blank microsome spiked with LB and IS and a real incubation sample (incubated for 30 min) were compared to check the separation and endogenous interferences.

##### Calibration curve

Calibration curve was obtained by plotting the area ratio (*y*) of peak of LB to that of IS against the concentration (*x*) of calibration curve samples. The linear regression was performed using 1/*x*^2^ as the weighting factor.

##### Accuracy and precision

Quality control samples were utilized to evaluate the accuracy and precision. Triplicate QC samples at each concentrate level were analyzed within 1 day for the intra-day assessment. Similarly, QC samples at each concentrate level were analyzed for three consecutive days for the determination of inter-day accuracy and precision. The accuracy was determined as the relative error (RE) between the measured and nominal concentrations while precision was calculated as the coefficient of variation (CV).

##### Recovery and stability

Recoveries of LB were determined by comparing the measured concentration of triplicate QC samples with triplicate samples prepared at equal concentration in precipitated matrix. The stability of LB in RLM was evaluated using QC samples after storage at room temperature for 24 h, three freeze-thaw cycles and storage at -80°C for 1 month.

### Optimizing of Incubation Conditions

Liver microsome from each individual rat was pooled equally for optimizing the incubation conditions.

#### Incubation Time

The volume of incubation system was 200 μL, containing 10 μL of microsome (final protein concentration was 1 mg/mL), 176 μL of PBS, 2 μL of LB (final concentration was 30 μg/mL) and 12 μL of NADPH-regenerating solution. The proportion of organic solvent was no more than 1%. Triplicate samples were preincubated at 37°C for 5 min prior to the addition of NADPH-regenerating solution and then the system was incubated at 37°C in a gentle shaking water-bath for 0, 5, 10, 20, 30, 45, 60, and 90 min followed by immediate transfer into ice-bath and preparation for HPLC-UV analysis. LB concentrations were quantified using the HPLC-UV method developed in the present study.

#### Protein Concentration

The volume of incubation system was 200 μL, containing 10 μL of microsome (final protein concentration was 0.2, 0.5, 1.0, 2.,0 and 5.0 mg/mL), 176 μL of PBS, 2 μL of LB (final concentration was 30 μg/mL) and 12 μL of NADPH-regenerating solution. The proportion of organic solvent was no more than 1%. Triplicate samples were preincubated at 37°C for 5 min prior to the addition of NADPH-regenerating solution and then the system was incubated at 37°C in a gentle shaking water-bath for 45 min followed by immediate transfer into ice-bath and preparation for HPLC-UV analysis. LB concentrations were quantified using the HPLC-UV method developed in the present study.

### Metabolic Features of LB in RLM

Liver microsome from each individual rat was pooled equally for the determination of metabolic features.

#### Metabolites Identification

The 200-μL incubation system was composed as follows: 10 μL of microsome (final protein concentration was 1 mg/mL), 176 μL of PBS, 2 μL of LB (final concentration was 30 μg/mL) and 12 μL of NADPH-regenerating solution. The proportion of organic solvent was no more than 1%. Samples were preincubated at 37°C for 5 min prior to the addition of NADPH-regenerating solution and then the system was incubated at 37°C in a gentle shaking water-bath for 45 min followed by immediate transfer into ice-bath. Two hundred microliters of ice-cold acetonitrile was added to terminate the reaction. Separation and identification of metabolites were performed using an LC-MS/MS method described in our previous study for detecting LB metabolites in human liver microsome (Li et al., 2017).

#### Metabolizing CYP450

Metabolizing CYP450 of LB was identified using the same incubation system as in the metabolites identification fortified with specific potent CYP450 inhibitors: α-naphthoflavone for CYP1A2, cimetidine for CYP2C11, quinindium for CYP2D1, 4-methylpyrazole for CYP2E1 and ketoconazole for CYP3A2 (Duhamel et al., 2010; Mishra et al., 2012; Xiang, 2013). The final concentrations of inhibitors were 100 μM ([*i*] >> *K_*i*_*) (Duhamel et al., 2010). Negative controls (without inhibitors) and positive controls (microsome protein was heat-denatured) were simultaneously tested. All the samples were performed in triplicate and LB concentrations were quantified using the HPLC-UV method developed in the present study. The strength of inhibition (*I*) was calculated as follows:

$$I\text{=}\frac{C_{\text{i}}-C_{\text{n}}}{C_{\text{p}}-C_{\text{n}}}\times \text{100}\%$$

Where *C_*i*_* was LB concentration in inhibitor sample, *C_*n*_* was LB concentration in negative control and *C_*p*_* was LB concentration in positive control.

#### Elimination Kinetics

Elimination kinetics was assessed using a similar incubation system as in the metabolites identification (except that the incubation time was 10 min) at varying final concentrations of LB ranging from 1.0 to 80 μg/mL. Samples were processed at 0 min (the addition of NADPH-regenerating solution) and at 10 min. Each concentration was performed in triplicate and LB concentrations were quantified using the HPLC-UV method developed in the present study. The initial velocity (*V_*0*_*) was approximated as follows:

$$V_{0}\text{=}\frac{C_{0}-C_{\text{t}}}{t\times C_{\text{protein}}}$$

Where *C_*0*_* was LB concentration at 0 min, *C_*t*_* was LB concentration at 10 min, *t* was time of incubation (10 min) and *C_*protein*_* was final concentration of microsome protein (1 mg/mL).

### Ability to Metabolize LB of Rat Liver

The abilities of livers to metabolize LB of 3-, 7-, 14-, and 21-day model groups were compared with those of respective control groups using the same incubation system as in the metabolites identification. The elimination amounts of LB were detected using the HPLC-UV method developed in the present study. Meanwhile, the relative generation amounts of LB metabolites were tested using the up-mentioned LC-MS/MS method (relative amounts were expressed as the ratio of mean metabolite peak area of model group to that of respective control group).

### Gene Expression of Major CYP450

#### RNA Isolation and qPCR Test

Total RNA was extracted from liver tissue using Trizol reagent according to the manufacturer’s protocol. cDNA was then prepared using the following method: 5 μg of total RNA was transferred into a 200-μL tube and mixed with 1 μL of Oligo(dT) 15 primer. RNase-free water was added to the mixture till the total volume was 14 μL. The RNA was denatured at 70°C for 5 min and then immediately put into ice-bath. 0.7 μL of RNasin Plus RNase Inhibitor, 5 μL of 5× M-MLV Reverse Transcriptase buffer, 1 μL of M-MLV Reverse Transcriptase, 1 μL of dNTPs and 3.3 μL of RNase-free water were orderly added to comprise the reverse transcription system. The system was incubated at 37°C for 1 h followed by 70°C for 5 min. The acquired cDNA was stored at -20°C until use. The qPCR test was performed using Bio-Rad iQ5 Real-Time PCR System (Bio-Rad, United States). Transcription levels of *CYP1A2, CYP2C11, CYP2D1, CYP2E1*, and *CYP3A2* were tested using SYBR^®^ Premix Ex Taq™ (Tli RNaseH Plus). Primers used were shown in **Table 1**. The fold change of mRNA level of individual CYP450 was calculated by normalizing the respective gene level to that of *GAPDH* using the 2^-ΔCt^ method.

**Table 1:** Primers used in the qPCR test.

Gene name	Primers
*CYP1A2*	F: 5′-TGCTACTTGTGACAGAGCCCAAG-3′
	R: 5′-ATCTCTGCCAATCACCGTGTCC-3′
*CYP2C11*	F: 5′-ACGTGGATGTCACAGCTAAAGTCC-3′
	R: 5′-GGCTCCGGTTTCTGCCAATTAC-3′
*CYP2D1*	F: 5′-ACAGCCTCTACAAGCTTCAACACC-3′
	R: 5′-ATGACCATGGGCTTCCAACCCTTC-3′
*CYP2E1*	F: 5′-ACAGCCATGAGTTTCCAGATCCAG-3′
	R: 5′-CTCCAACACACACACGCTTTCCTG-3′
*CYP3A2*	F: 5′-TCCTGGCCACTCACCCTGATATTC-3′
	R: 5′-TCGTAGGTAGGAGGTGCCTTACTC-3′
*GAPDH*	F: 5′-TCTCTTGTGACAAAGTGGACAT-3′
	R: 5′-GGTGATGGGTTTCCCGTTGA-3′

#### Western Blot Assay

For Western blot, 0.5 g of liver tissue was put into 3.5 mL of ice-cold RIPA (containing cocktail protease inhibitor, Roche Diagnostics, Switzerland) and homogenized thoroughly. The homogenate was then centrifuged at 12,000 ×*g* for 10 min at 4°C to collect total liver protein. After denatured using protein loading buffer, protein samples were loaded onto a 12% SDS-PAGE and transferred to a PVDF membrane after the electrophoresis. The membrane was then blocked by 5% non-fat milk in TBST for 2 h at room temperature. Incubation of the membrane with primary antibodies of CYP1A2, CYP2C11, CYP2D1, CYP2E1, CYP3A2, and GAPDH was performed at 4°C overnight. Next, the membrane was washed with TBST four times and then incubated with HRP-conjugated secondary antibody for 2 h at room temperature. After the second 4-time wash in TBST, the membrane was developed using ECL plus Western blot detection system. GAPDH protein level was used as the loading control.

### Statistical Analysis

SPSS 20.0 software (IBM, United States) was used to perform statistical analysis. Difference between groups was tested by Independent-Samples *t*-test. A *p*-value less than 0.05 was considered to be statistically significant. Non-linear regression for elimination kinetics was executed by GraphPad Prism 6 software (GraphPad Software, United States).

## Results and Discussion

Many *in vivo* studies of LB have been performed by our group, including plasma pharmacokinetics, excretion, and bioavailability. Two phase II metabolites of LB were identified in rats’ plasma, bile and urine using an LC-MS/MS method (unpublished data). However, no obvious phase I metabolites were identified for now. Hence, it is of our interest to investigate the metabolic features of LB in RLM, including metabolites identification, elimination kinetics and metabolizing CYP450. Furthermore, since previous studies of our group reported that SM obviously decreased plasma concentration of LB (Li et al., 2014; Chen et al., 2017) and metabolism is a crucial step of pharmacokinetics, we also investigated the effects of SM on LB metabolism in the present study.

### The HPLC-UV Method

**FIGURE 2:**
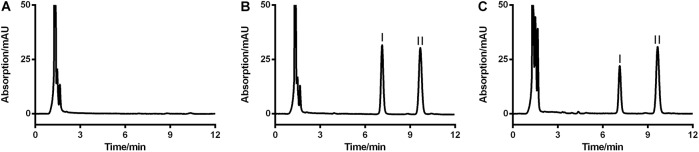
Representative HPLC-UV chromatograms of blank RLM **(A)**, blank RLM spiked with 20 μg/mL of LB and 6.7 μg/mL of IS **(B)**, and a real incubation sample (**C**, 20 μg/mL of LB was incubated for 30 min). LB and IS were successfully separated and no obvious endogenous interference existed. Peaks I and II were LB and IS, respectively.

Since no applicable bio-analytical method was previously reported, we initially developed a novel HPLC-UV method for quantifying LB in RLM. The chromatographic conditions were optimized by the investigations of columns, mobile phase composition and measure wavelength. As illustrated in **Figure 2**, LB and IS were successfully separated on the HPLC system and no obvious endogenous interferences were observed. The retention time of LB and IS were 7.1 and 9.7 min, respectively. The calibration curve was constructed by eight concentration points (1.0–100 μg/mL). A typical regression equation was *y* = 0.0414*x* + 0.0073 with coefficient of correlation *r* > 0.99. The limit of quantification was 1.0 μg/mL whose accuracy and precision were 2.0% and 4.1%, determined using six replicate samples and the sensitivity was adequate for the study. Accuracy, precision and recovery results using QC samples were presented in **Table 2**. The stability for investigated conditions was between -13.7% and 0.74%. All results were within the acceptable limitations (Bansal and DeStefano, 2007).

**Table 2:** Accuracy, precision, and recovery for quantification of LB in RLM.

QC samples (μg/mL)	Accuracy (RE, %)	Precision (RSD, %)		Recovery (%)
		Intra-day	Inter-day	
Low (2.0)	1.4	3.4	7.5	108.0
Medium (20)	1.4	4.2	4.4	106.3
High (80)	-11.2	6.5	0.5	91.9

### The Optimal Incubation Conditions

**FIGURE 3:**
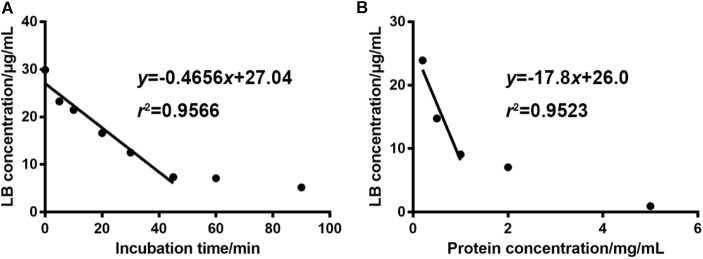
For 30 μg/mL of LB, the optimal incubation time and RLM protein concentration were 45 min **(A)** and 1 mg/mL **(B)**, respectively. (mean ± SD, *n* = 3).

Optimal incubation milieu is crucial for the evaluation of metabolic features. Long incubation time within the linear elimination range can singularize the metabolism and thus simplify the data interpretation (Zhang et al., 2015). Appropriate microsome protein concentration will minimize the non-specific bindings of enzymes and substrates (Jones and Houston, 2004). So, the incubation time and the dosage of liver microsome protein were optimized right after the development of the HPLC-UV method. **Figure 3A** showed that when the concentration of RLM protein was fixed at 1 mg/mL in the incubation system, LB was linearly eliminated from 29.87 μg/mL at 0 min to 7.34 μg/mL at 45 min. After the linear elimination, concentration of LB showed no obvious decrease (to 5.20 μg/mL at 90 min). **Figure 3B** showed that 1 mg/mL was the best protein concentration for the 45-min incubation as the elimination of LB was not significantly increased when RLM protein was more added. Hence, the incubation time and the microsome protein concentration used in the present study (except for the incubation time in elimination kinetics) were set at 45 min and 1 mg/mL, respectively.

### LB Metabolites in RLM

**FIGURE 4:**
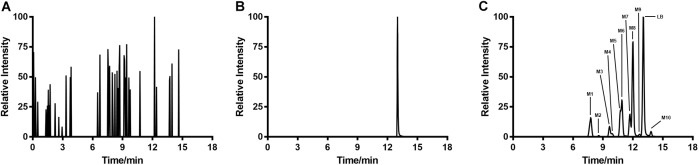
Extracted ion chromatograms of blank RLM **(A)**, blank RLM spiked with 30 μg/mL of LB **(B)**, and a real incubation sample (**C**, 30 μg/mL of LB was incubated for 45 min). Ten metabolites in total (M1–M10) were suggested.

**FIGURE 5:**
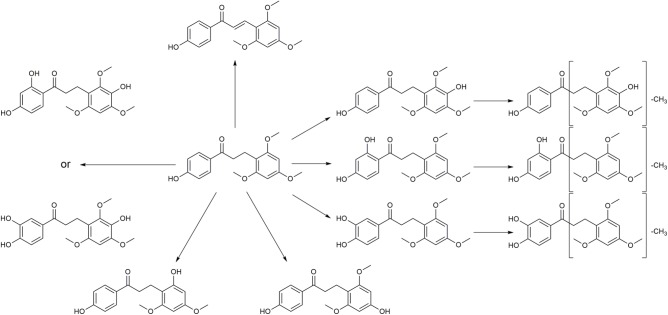
Proposed metabolic pathways of LB in RLM.

Typical extracted ion chromatograms of blank RLM, blank RLM spiked with LB and real incubation samples were shown in **Figure 4**, by which 10 metabolites (M1–M10) were suggested. Fragmentation pathway of LB (**Supplementary Figure S1**) previously reported (Li et al., 2017) was used to tentatively deduce chemical structures of metabolites based on their fragmentation pathways (**Supplementary Figure S2**). Brief information of metabolites was listed in **Table 3** and metabolic pathways of LB in RLM were shown in **Figure 5**.

**Table 3:** Brief information of LB metabolites in RLM.

	Molecular formula	Calculated mass	Measured mass	Error (ppm)	Metabolic type
M1	C_17_H_19_O_6_	319.1176	319.1173	-0.94	Demethylation and hydroxylation
M2	C_17_H_19_O_6_	319.1176	319.1174	-0.63	Demethylation and hydroxylation
M3	C_17_H_19_O_6_	319.1176	319.1171	-1.57	Demethylation and hydroxylation
M4	C_18_H_21_O_7_	349.1282	349.1276	-1.72	Di-hydroxylation
M5	C_17_H_19_O_5_	303.1227	303.1220	-2.31	Demethylation
M6	C_18_H_21_O_6_	333.1333	333.1326	-2.10	Hydroxylation
M7	C_17_H_19_O_5_	303.1227	303.1219	-2.64	Demethylation
M8	C_18_H_21_O_6_	333.1333	333.1325	-2.40	Hydroxylation
M9	C_18_H_19_O_5_	315.1227	315.1220	-2.22	Reduction
M10	C_18_H_21_O_6_	333.1333	333.1325	-2.40	Hydroxylation

M4 showed the [M + H]^+^ ion at m/z 349.1, 32 Da (two oxygen atoms) more than that of LB, suggesting that M4 was a di-hydroxylated metabolite. Fragment ion at m/z 197.1 (**Supplementary Figure S2.1**), the hydroxylated [LB + H-C_8_H_8_O_2_]^+^ fragment, indicating that one hydroxylation occurred on B ring and the other on A ring. M6, M8, and M10 all gave the same [M + H]^+^ ion at m/z 333.1, 16 Da (one oxygen atom) heavier than that of LB, suggesting that M6, M8, and M10 were three mono-hydroxylated metabolites. The [LB + H-C_8_H_8_O_2_]^+^ fragment of M6 was hydroxylated while those of M8 and M10 were not (**Supplementary Figure S2.2**). Hence, M6 was inferred as B ring hydroxylated metabolite while M8 and M10 were A ring hydroxylated metabolites.

M1, M2, and M3 all exhibited the same [M + H]^+^ ion at m/z 319.1, 14 Da (CH_2_) less than the mono-hydroxylated metabolites, suggesting that they were demethylated products of mono-hydroxylated LB. The demethylated hydroxylated [LB + H-C_8_H_8_O_2_]^+^ fragment ion at m/z 183.1 of M3 indicated that demethylation and hydroxylation both happened on B ring (demethylated M6). Though M1 and M2 showed different fragmentation patterns, we deduced that they were demethylated products of M8 and M10 (**Supplementary Figure S2.3**).

M5 and M7 both gave the same [M + H]^+^ ion at m/z 303.1, 14 Da (CH_2_) less than LB, suggesting that they were demethylated metabolites. The identical fragment ion [LB + H-C_8_H_8_O_2_-CH_2_]^+^ at m/z 167.1 of M5 and M7 demonstrated that the demethylation was induced on B ring (**Supplementary Figure S2.4**). Additionally, M9 showing the [M + H]^+^ ion at m/z 315.1, 2 Da (two hydrogen atoms) less than LB, was considered to be the oxidized metabolite. Though the exact structure of M9 was difficult to be inferred from the fragment ions (**Supplementary Figure S2.5**), the oxidation site was proposed at C_α_–C_β_ single bond (Li et al., 2017).

### Metabolizing CYP450 of LB in RLM

**FIGURE 6:**
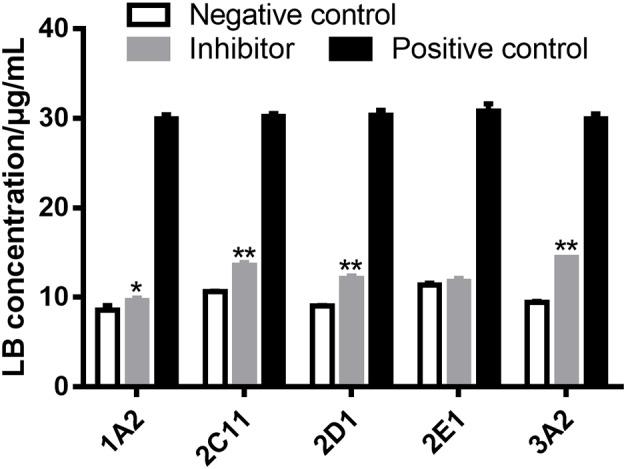
Elimination of LB was significantly inhibited by α-naphthoflavone, cimetidine, quinindium, or ketoconazole indicating that CYP1A2, CYP2C11, CYP2D1, and CYP3A2 were involved in the metabolism of LB. ^∗^*p* < 0.05, ^∗∗^*p* < 0.01, compared with negative control (mean ± SD, *n* = 3).

Several strategies are used to identify the CYP450 involved in the metabolism of a drug, such as potent selective inhibitors, specific antibodies and recombinant CYP450 isoforms (Venkatakrishnan et al., 2001). The inhibitor method is well accepted for its low cost, availability and safety (Lu et al., 2003). Inhibitors employed in the examination of metabolizing CYP450 of LB in RLM were α-naphthoflavone for CYP1A2, cimetidine for CYP2C11, quinindium for CYP2D1, 4-methylpyrazole for CYP2E1 and ketoconazole for CYP3A2, respectively. As shown in **Figure 6**, the addition of α-naphthoflavone, cimetidine, quinindium or ketoconazole remarkably reduced the elimination of LB indicating the involvements of CYP1A2, CYP2C11, CYP2D1, and CYP3A2 in LB metabolism. The relative strength was: CYP3A2 (24.1%) > CYP2C11 (15.0%) > CYP2D1 (14.4%) > CYP1A2 (4.8%).

The results of metabolizing CYP450 tests demonstrated that at least four major CYP450 isoforms were responsible for the metabolism of LB, which was noticeable considering that DB might be co-administered with other drugs also eliminated by CYP450. For example, promethazine, the standard agent for space motion sickness (Paule et al., 2004), is metabolized by CYP2D (Litzenburger et al., 2015); zolpidem, one of the medications for sleep promotion (Shi et al., 2003), is metabolized by CYP3A (Hesse et al., 2003) and ibuprofen, used for pain relief during spaceflight (Graebe et al., 2004), is metabolized by CYP2C (Krasniqi et al., 2016). Hence, the co-administration of DB with other drugs might lead to potential drug–drug interaction and adverse effects, which should be fully predicted. Furthermore, previous studies suggested that CYP1A, CYP2C, CYP2D, and CYP3A were subject to polymorphisms (Preissner et al., 2013). Consequently, inter-individual variability in the clearance of LB should be adequately investigated to avoid therapeutic failure or unexpected toxicity (Deenen et al., 2011).

### Elimination Kinetics of LB in RLM

**FIGURE 7:**
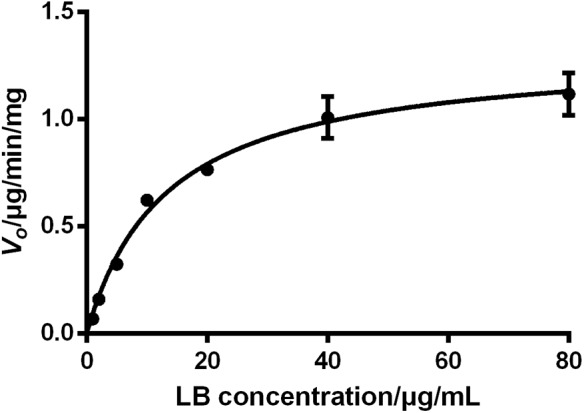
Elimination kinetics of LB in RLM followed a typical Michaelis–Menten model with *K*_*m*_ of 13.33 μg/mL and *V*_*max*_ of 1.32 μg/min/mg (mean ± SD, *n* = 3).

The sufficient understanding of pharmacokinetic and metabolic features of a drug requires the determination of enzyme–substrate interactions (Duhamel et al., 2010; Mishra et al., 2012). Enzyme kinetics can be usually assessed by measuring the product formation rate at varying concentrations of substrate. Since the identification of metabolites of a chemical is time-consuming, monitoring the disappearance rate of substrate is also considered as an acceptable approach in the preliminary stage (Duhamel et al., 2010; Mishra et al., 2012). The period of incubation in enzyme kinetics test should be short to reflect the transient loss of substrates and thus the *V_*0*_* of the reaction can be approximated. Therefore, the *V_*0*_* of LB elimination in RLM was calculated using measured concentration of LB at 0 min and 10 min tested by the HPLC-UV method. The results were fitted to a typical Michaelis–Menten model with a regression coefficient *r* > 0.99, as illustrated by **Figure 7**. The kinetic parameters were *K*_*m*_ of 13.33 μg/mL, *V*_*max*_ of 1.32 μg/min/mg, and *Cl_*int*_* (*V*_*max*_/*K*_*m*_) of 99.02 μL/min/mg.

The effect of substrate concentrations on the *V_*0*_* of an enzyme-catalyzed reaction plays a dominant role in the enzyme kinetics. It is well accepted that CYP450 does not always conform to typical Michaelis–Menten processes and some isoforms exhibit several kinds of allosteric kinetics (Atkins, 2004). CYP450 in RLM generate multiple products from LB (M1–M10) and this ‘one substrate to multiple products’ biotransformation can significantly complicate the enzyme–substrate interactions (Atkins, 2004). Hence, the *K*_*m*_ and *V*_*max*_ obtained from the present study should be considered as apparent parameters and therefore, further investigations should be conducted using purified CYP450 isoforms to test enzyme kinetics for each product (Messiano et al., 2013).

### Effects of SM on LB Metabolism in RLM

**FIGURE 8:**
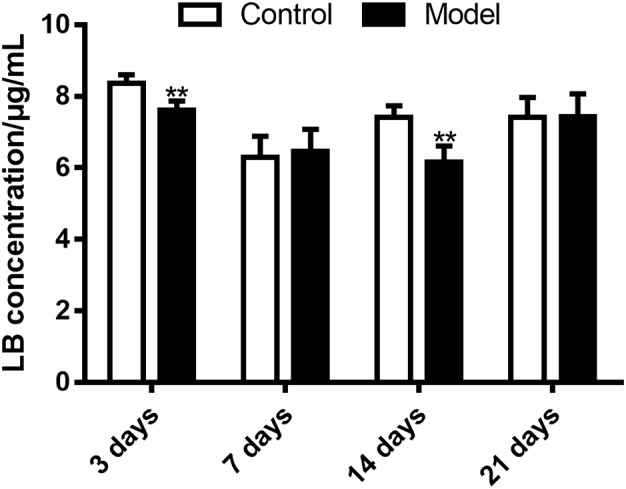
Elimination of LB in RLM remarkably increased after 3-day and 14-day SM. ^∗∗^*p* < 0.01, compared with control group (mean ± SD, *n* = 6).

The HPLC-UV method and the optimal incubation conditions were then applied in investigating the effects of SM on LB elimination. The elimination amounts were indirectly compared using the epibiotic concentration of LB in RLM prepared from control and model group rats. As illustrated in **Figure 8**, the effects varied with the duration of SM. After 3-day and 14-day SM, LB concentration left in RLM of control groups after the 45-min incubation marked 8.36 ± 0.25 and 7.42 ± 0.32 μg/mL, respectively, while those of model groups significantly decreased to 7.61 ± 0.26 and 6.16 ± 0.46 μg/mL (*p* < 0.01, compared with control groups). LB concentration of other durations showed no obvious alterations.

**FIGURE 9:**
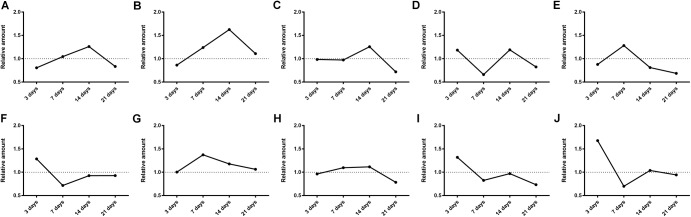
Generation of LB metabolites, especially after 3-day and 14-day SM was obviously disrupted. **(A–J)** were results for M1–M10, respectively. Data was shown as the ratio of mean metabolite peak area of model group to that of respective control group (*n* = 6).

Then the effects of SM on the generation amounts of LB metabolites were tested using the LC-MS/MS method. Since the reference substances of metabolites were not available for now, a relative quantification strategy was employed, that was, the relative generation amounts in model groups were normalized by those of control groups. As illustrated in **Figure 9**, the effects were complex and varied with the duration of SM. For example, after 3-day SM, production of M4, M6, and M10, three hydroxylated metabolites and M9, the oxidized metabolite in model group was increased by 1.2-, 1.3-, 1.7-, and 1.3-fold, respectively; M3, M7, and M8 showed no obvious difference between the two groups; only M1, M2, and M5 were scarcely less generated in model group (no less than 80% of those in control group). After 14-day SM, totally 6 of the 10 metabolites (M1, M2, M3, M4, M7, and M8) were sharply accumulated in model group.

The elimination amounts of LB in RLM were demonstrated to be increased by 3-day and 14-day SM, which might correspondently decrease the pharmacological effects of DB. Hence, does adjustments would be essential if DB is applied in missions. To our knowledge, it was the first time that RLM from rats in SM condition was used for the prediction of drug metabolism in microgravity, which might provide some enlightenment for the future work. The elimination amounts of LB represented the integral metabolic ability of RLM. In order to explore the elaborate alterations on LB metabolism in SM condition, the generation amounts of metabolites was then studied. The obvious up-regulation of M4, M6, M9, and M10, together with the slight down-regulation of M1, M2, and M5 might synthetically contribute to the increased elimination of LB of 3-day model group. And the sharp accumulation of M1, M2, M3, M4, M7, and M8 in 14-day model group might consume LB heavily as compared with the control.

### Effects of SM on Gene Expression of Major CYP450

**FIGURE 10:**
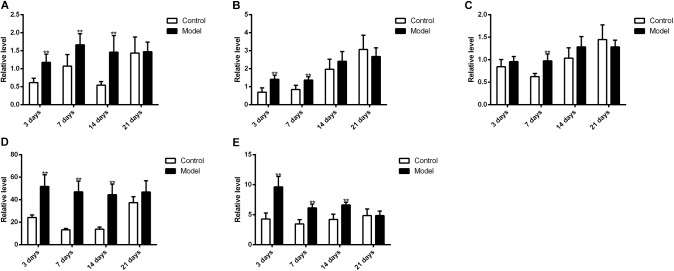
qPCR results for transcription levels of major CYP450 genes. ^∗∗^*p* < 0.01, compared with control group (mean ± SD, *n* = 6).

**FIGURE 11:**
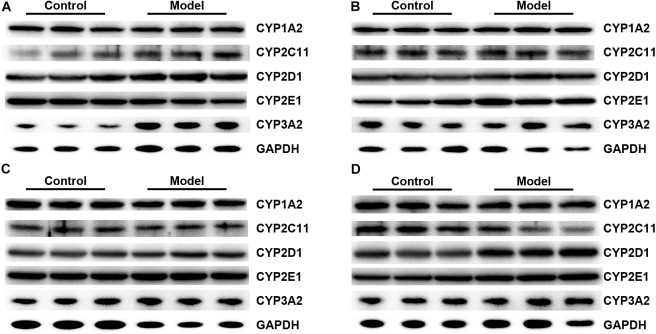
Representative Western blot bands for 3-day **(A)**, 7-day **(B)**, 14-day **(C)**, and 21-day SM **(D)**.

**FIGURE 12:**
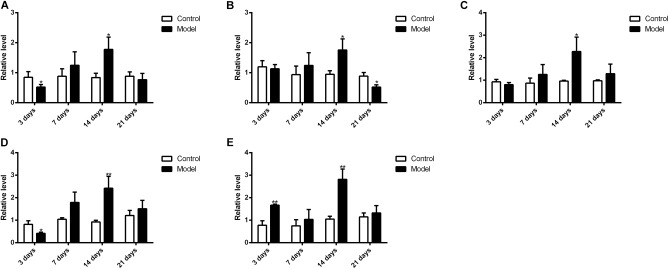
Western blot results for protein levels of major CYP450. ^∗^*p* < 0.05, ^∗∗^*p* < 0.01, compared with control group (mean ± SD, *n* = 6).

**Figure 10** illustrated that the regulatory effects of SM on the mRNA levels depended on isoforms and time courses. The transcription of *CYP1A2, CYP2E1*, and *CYP3A2* genes was persistently and obviously induced after 3-, 7-, and 14-day SM. For example, mRNA levels of *CYP2E1* were increased by more than twofold at all time points (*p* < 0.01, compared with control groups). The amounts of mRNA of *CYP2C11* and *CYP2D1* genes were only up-regulated by short-term SM. 3-day SM increased transcription of *CYP2C11* by about twofold (*p* < 0.01, compared with control group), whereas *CYP2D1* was not changed. 7-day SM, respectively, led to 1.6-fold increase in mRNA contents of *CYP2C11* and *CYP2D1* (*p* < 0.01, compared with control groups). No significant changes were captured after 21-day SM. Similarly, protein contents of CYP450 were differentially regulated by various SM durations, as shown in **Figures 11, 12** (original Western blot images were shown in **Supplementary Data Sheet S1**). The most noticeable alterations were captured after 14-day SM when all the five isoforms were sharply up-regulated (*p* < 0.05 or 0.01, compared with control groups). 3-day SM triggered different changes in the amounts of the investigated enzymes. CYP1A2 and CYP2E1 were both down-regulated significantly by 3-day SM (*p* < 0.05, compared with control group) while CYP3A2 was increased (*p* < 0.01, compared with control group). Long-term SM only suppressed the expression of CYP2C11 (*p* < 0.05, compared with control group).

Previous studies have suggested that the expression of CYP450 is mainly regulated by nuclear receptors, such as constitutive androstane receptor, pregnane X receptor, aryl hydrocarbon receptor and so on (Maglich et al., 2003; Faucette et al., 2006; Haduch et al., 2007; Rhodes et al., 2011). For example, after treated with 400 mg/kg nano-copper, the expression of CYP1A2, CYP2C11, CYP2E1, and CYP3A2 of rats’ liver significantly decreased with the suppression of constitutive androstane receptor, pregnane X receptor and aryl hydrocarbon receptor (Tang et al., 2018); decreased expression of CYP1A2, CYP2D6, and CYP2E1 of human liver was also associated with downregulation of aryl hydrocarbon receptor in liver fibrosis (Hanada et al., 2012). However, hepatic content of nuclear receptor is barely studied in either real spaceflight or SM. It is of great importance to investigate the mechanism of CYP450 regulation in microgravity condition in the future. Meanwhile, variations of hepatic CYP450 levels might help to understand the changes of drug metabolism in liver. 3-day and 14-day SM exerted obvious effects on the protein levels of CYP1A2, CYP2D1, CYP2E1, and CYP3A2 while 14-day and 21-day SM affected the content of CYP2C11 significantly. CYP1A2, CYP2C11, CYP2D1, and CYP3A2 were suggested to be involved in the metabolism of LB so the sharp up-regulation of the four isoforms after 14-day SM could contribute to the increased elimination of LB in RLM. The increased elimination after 3-day SM might be caused by the induction of CYP3A2 as the enzyme dominated the metabolism of LB. To our knowledge, it was the first time to determine the effects of SM on the mRNA and protein levels of major CYP450 in rat liver using qPCR and Western blot tests. As mentioned above, some widely used medications in manned spaceflight, such as promethazine, zolpidem, and ibuprofen, are substrates of CYP450. Hence, the alterations of major CYP450 contents should be taken into account if dose adjustments are performed for astronauts in real microgravity.

## Conclusion

In summary, 10 metabolites of LB in RLM were reported in the present study. At least four major CYP450 isoforms participated in the metabolism of LB and the elimination kinetics complied with a typical Michaelis–Menten equation. SM increased the hepatic elimination of LB in rats though the effect depended on time course. Additionally, generation of LB metabolites was also altered by SM. Furthermore, the expression of five most abundant CYP450 isoforms in rat liver was dramatically affected by SM, which might be an important factor for the altered metabolism of LB. The results might provide supports for the application of DB and enlightenment for the reasonable use of current medications metabolized by CYP450 in space missions.

Though it possesses multiple pharmacological activities and is used for the QC, LB is just one component of DB. Hence, the metabolic features of other bio-active compounds should be investigated for the further research of DB. And metabolic variations of commonly used medications for astronauts can be determined using the method provided in the current study. Moreover, the Morey-Holton rat model is a ground-based model of microgravity and future work can be performed in real spaceflight to study the drug metabolism in the space.

## Author Contributions

YD and YL: research design. BC, JG, SW, and LK: experiment execution. BC: manuscript writing.

## Conflict of Interest Statement

The authors declare that the research was conducted in the absence of any commercial or financial relationships that could be construed as a potential conflict of interest.

## Supplementary Material

FIGURE S1 Fragmentation pathways of LB under the LC-MS/MS conditions which was tested in a previous study of our group (Li et al., 2017).

FIGURE S2.1 MS^2^ spectrum of M4.

FIGURE S2.2 MS^2^ spectrum of M6, M8, and M10.

FIGURE S2.3 MS^2^ spectrum of M1, M2, and M3.

FIGURE S2.4 MS^2^ spectrum of M5 and M7.

FIGURE S2.5 MS^2^ spectrum of M9.

DATA SHEET S1 Original Western blot images.

## Abbreviations

CYP450: cytochrome P450
DB: dragon blood
IS: internal standard
LB: loureirin B
QC: quality control
RLM: rat liver microsome
SM: simulated microgravity
