# Supplementary material for: Simulated Microgravity Altered the Metabolism of Loureirin B and the Expression of Major Cytochrome P450 in Liver of Rats
Source: Front Pharmacol. 2018 Oct 12;9:1130. doi: 10.3389/fphar.2018.01130 (PMC6194197; doi:10.3389/fphar.2018.01130)
Supplement: FIGURE S1 — Fragmentation pathways of LB under the LC-MS/MS conditions which was tested in a previous study of our group (Li et al., 2017). [file Table_1.doc]

Supplementary Material

# Simulated Microgravity Altered the Metabolism of Loureirin B and the Expression of Major Cytochrome P450 in Liver of Rats

Bo Chen^1^, Jingjing Guo^1^, Shibo Wang^1^, Liting Kang^1^, Yulin Deng^1*^, Yujuan Li^1*^

*** Correspondence:**

Yulin Deng, Yujuan Li

[Deng@bit.edu.cn](mailto:Deng@bit.edu.cn), [lylyjlzh2006@163.com](mailto:lylyjlzh2006@163.com)


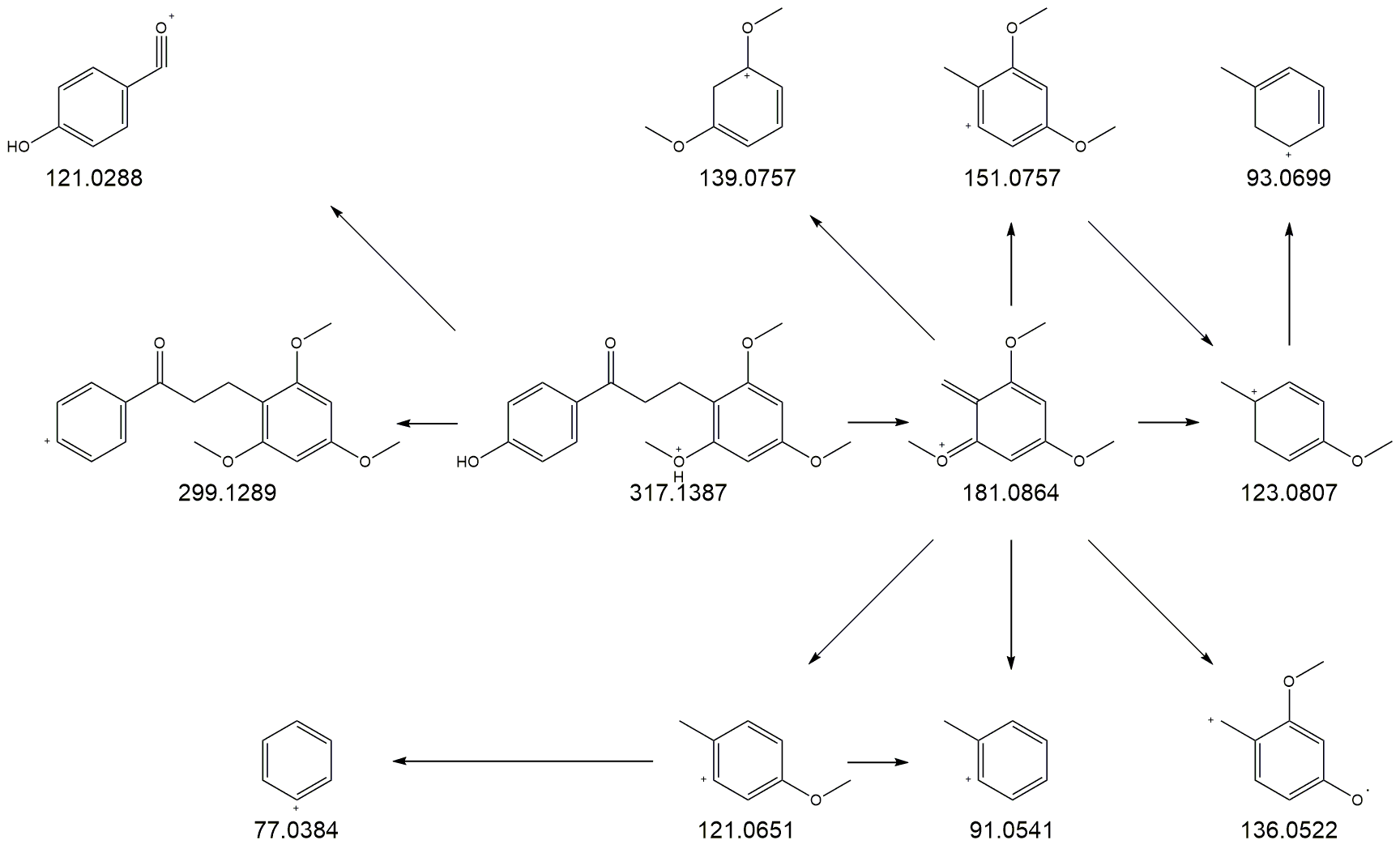


**Supplementary Figure 1** Fragmentation pathways of LB under the LC-MS/MS conditions which was tested in a previous study of our group .

Li, Y., Zhang, Y., Wang, R., Wei, L., Deng, Y., and Ren, W. (2017). Metabolic profiling of five flavonoids from Dragon's Blood in human liver microsomes using high-performance liquid chromatography coupled with high resolution mass spectrometry. *J Chromatogr B Analyt Technol Biomed Life Sci* 1052**,** 91-102. doi: 10.1016/j.jchromb.2017.03.022.
