# Supplementary material for: Simulated Microgravity Altered the Metabolism of Loureirin B and the Expression of Major Cytochrome P450 in Liver of Rats
Source: Front Pharmacol. 2018 Oct 12;9:1130. doi: 10.3389/fphar.2018.01130 (PMC6194197; doi:10.3389/fphar.2018.01130)
Supplement: FIGURE S2.1 — MS2 spectrum of M4. [file Table_2.doc]

Supplementary Material

# Simulated Microgravity Altered the Metabolism of Loureirin B and the Expression of Major Cytochrome P450 in Liver of Rats

Bo Chen^1^, Jingjing Guo^1^, Shibo Wang^1^, Liting Kang^1^, Yulin Deng^1*^, Yujuan Li^1*^

*** Correspondence:**

Yulin Deng, Yujuan Li

[Deng@bit.edu.cn](mailto:Deng@bit.edu.cn), [lylyjlzh2006@163.com](mailto:lylyjlzh2006@163.com)

**Supplementary Figure 2.1** MS^2^ spectrum of M4.


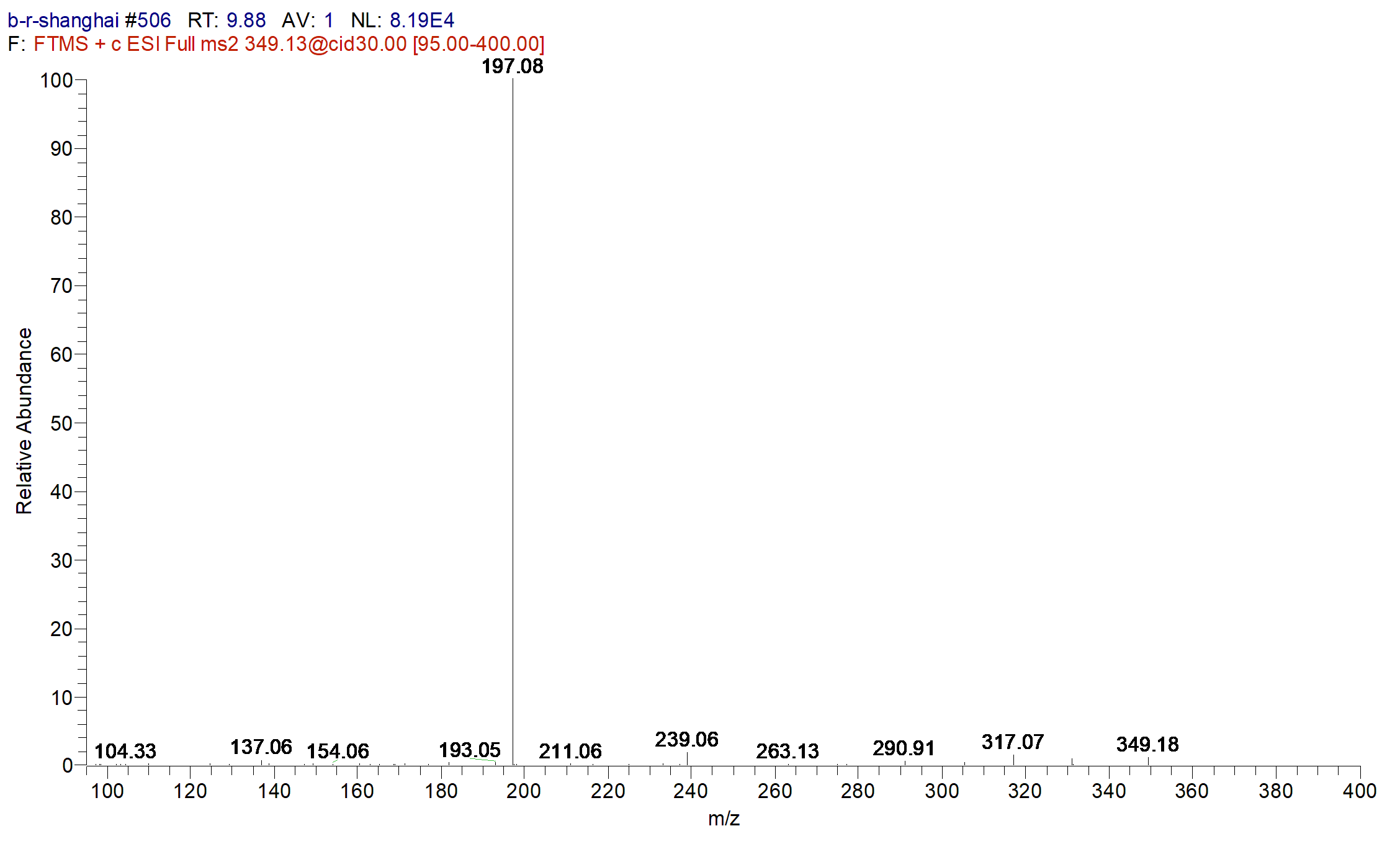

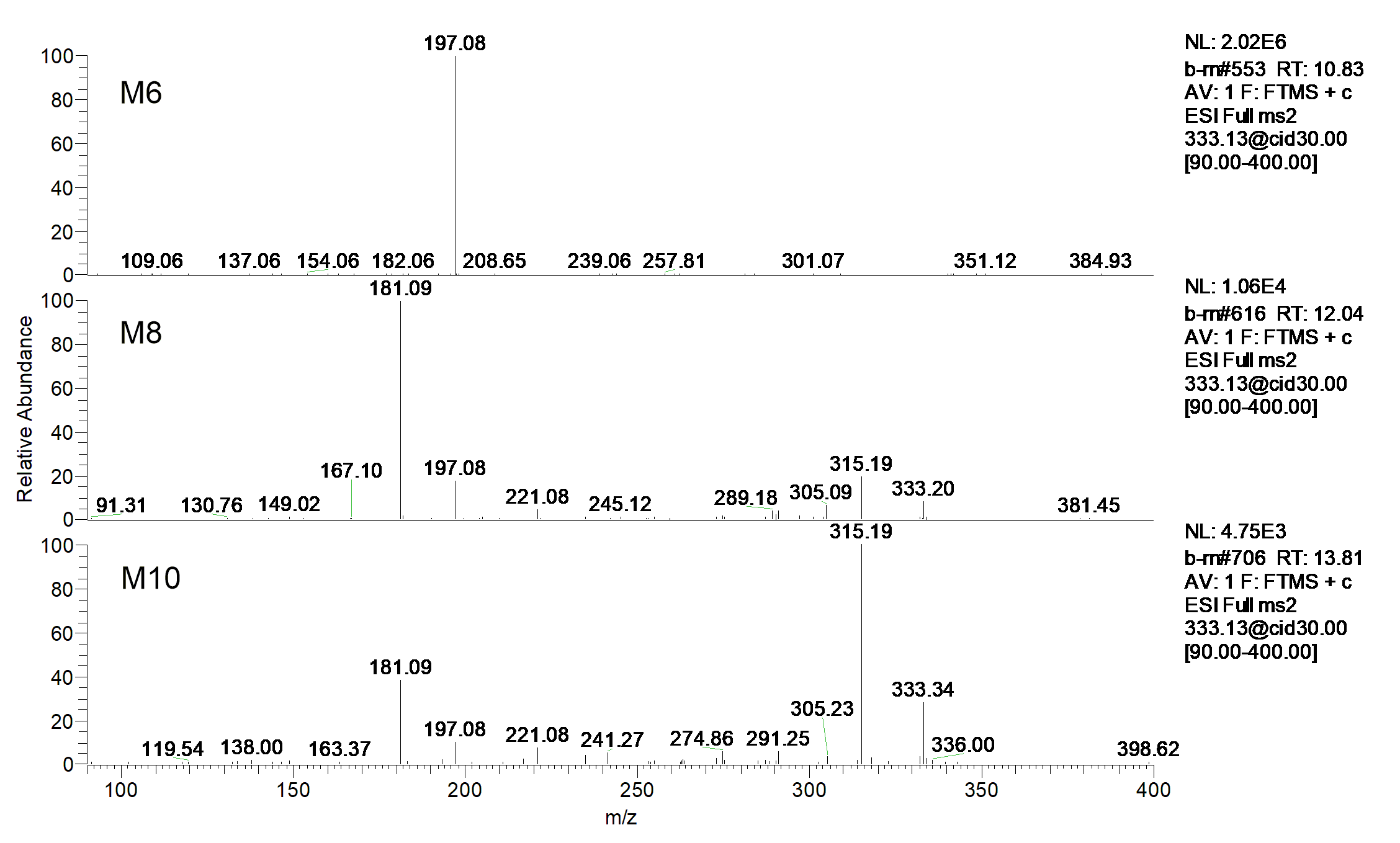


**Supplementary Figure 2.2** MS^2^ spectrum of M6, M8 and M10.


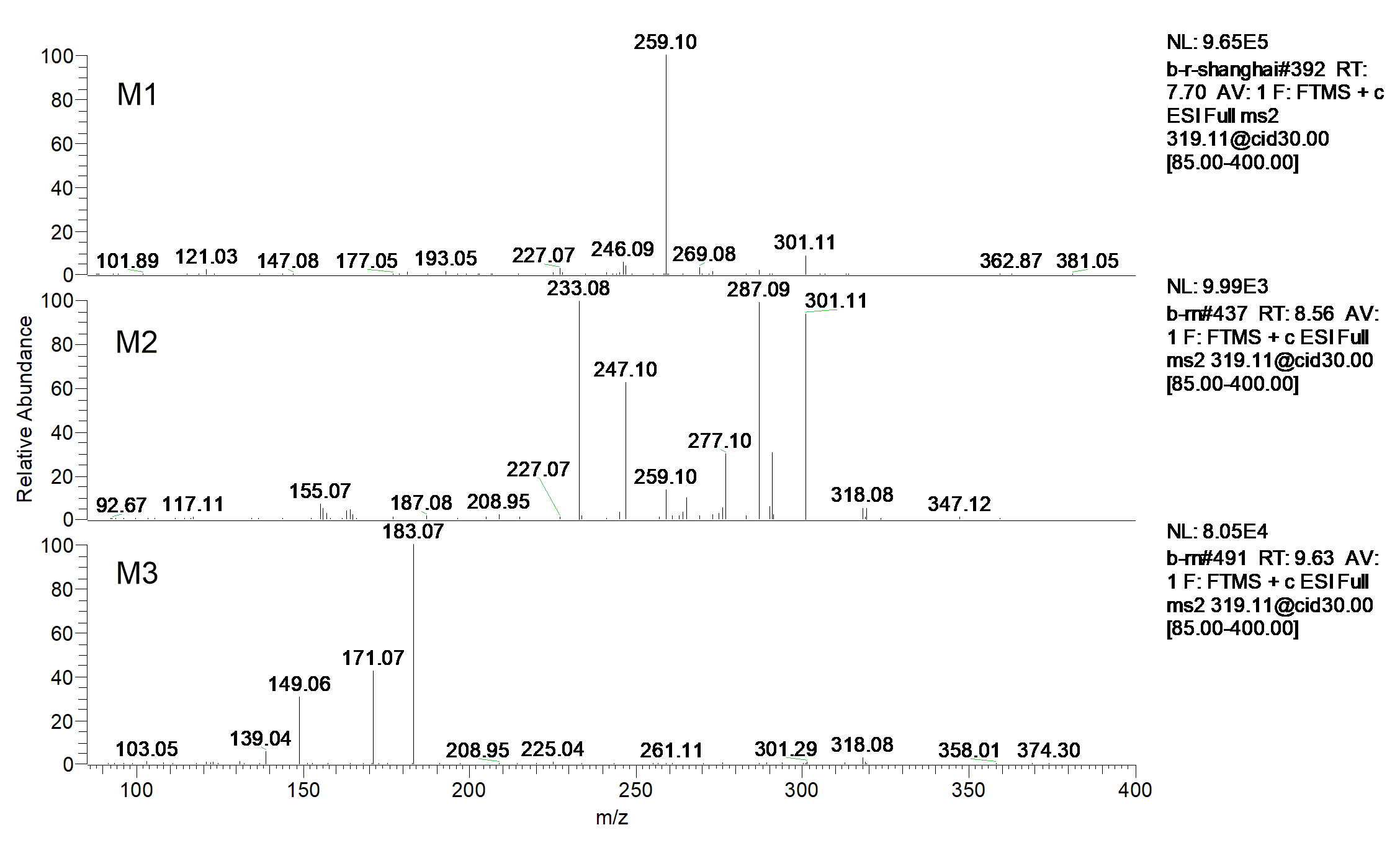


**Supplementary Figure 2.3** MS^2^ spectrum of M1, M2 and M3.

**Supplementary Figure 2.4** MS^2^ spectrum of M5 and M7.


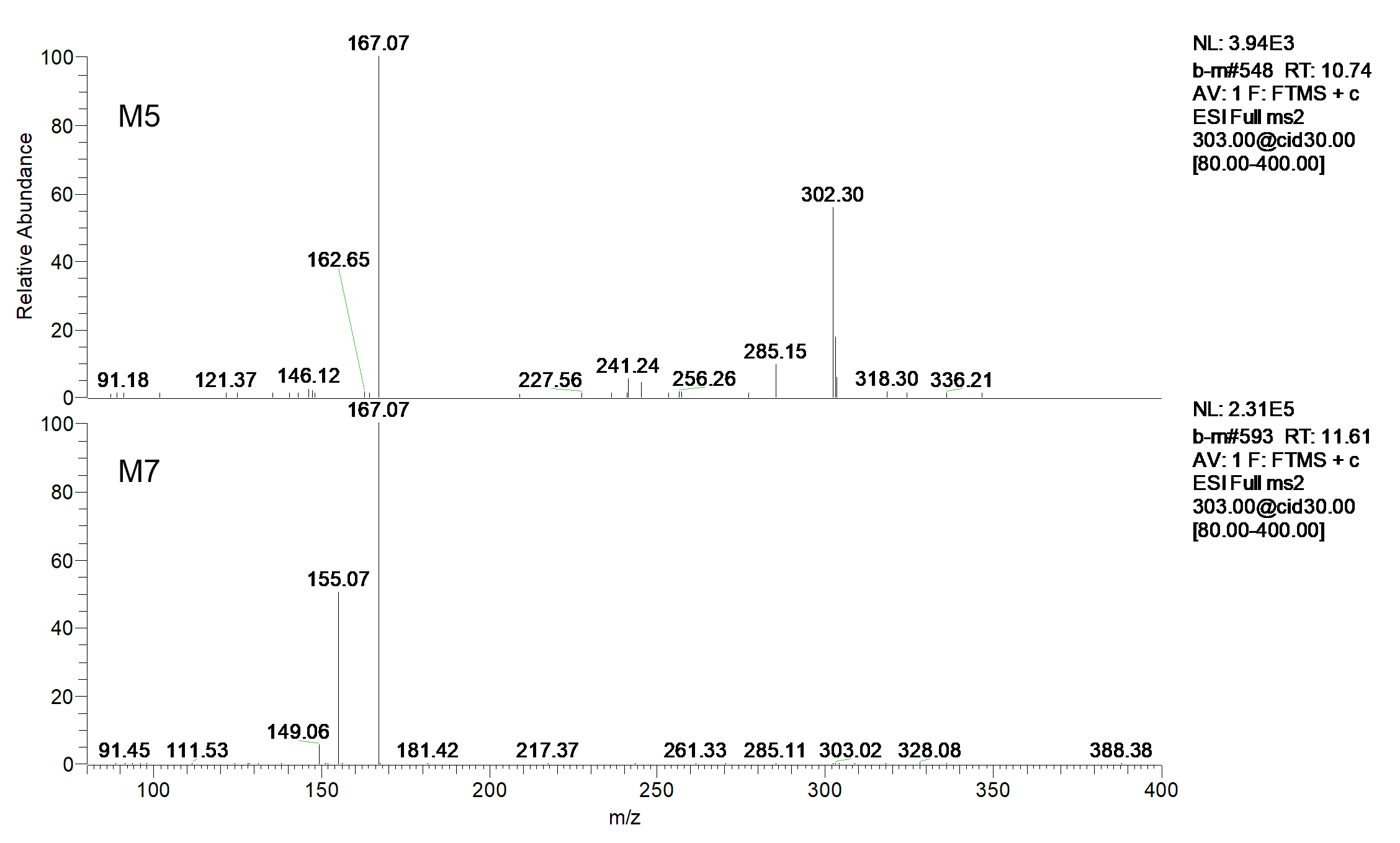

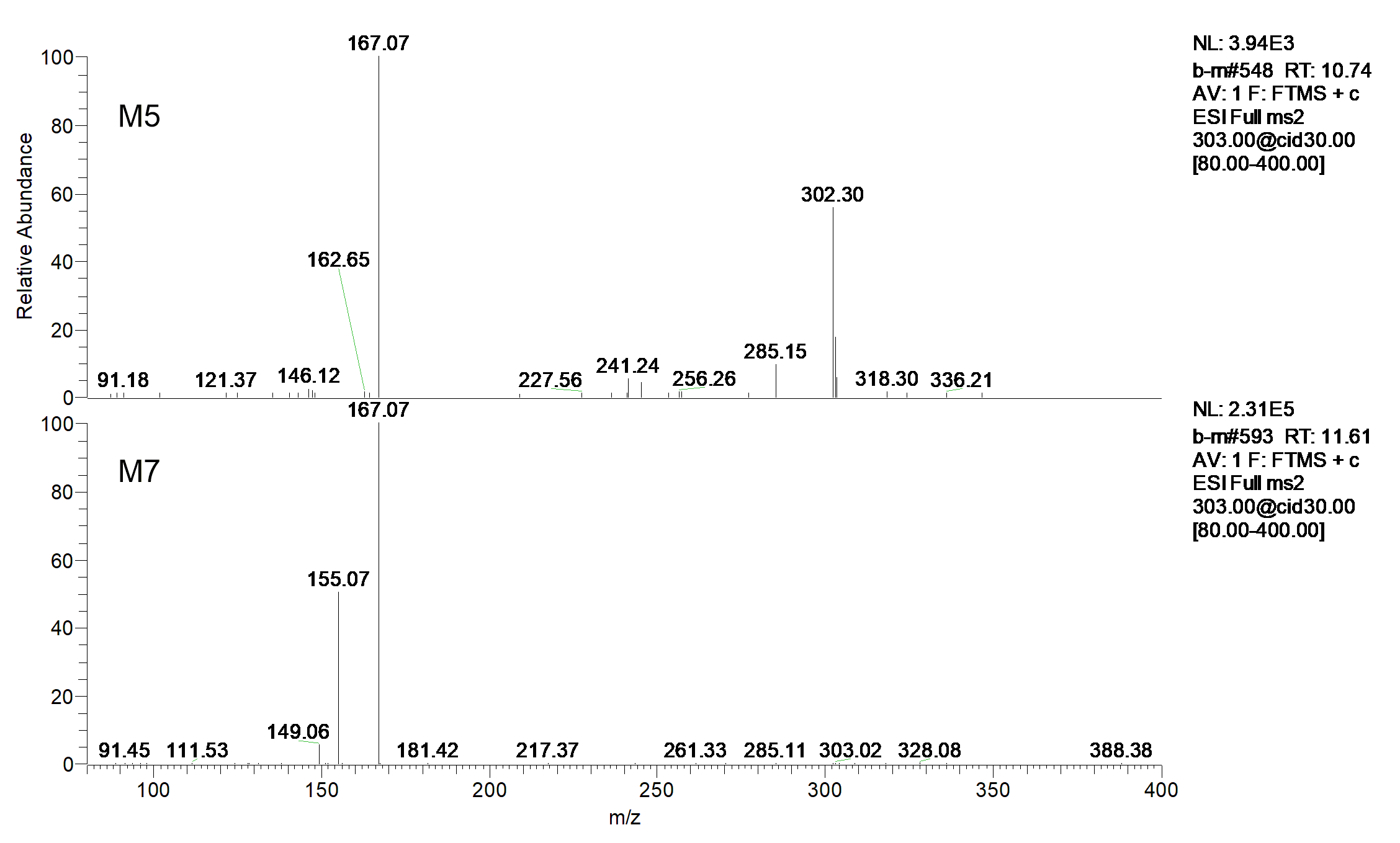

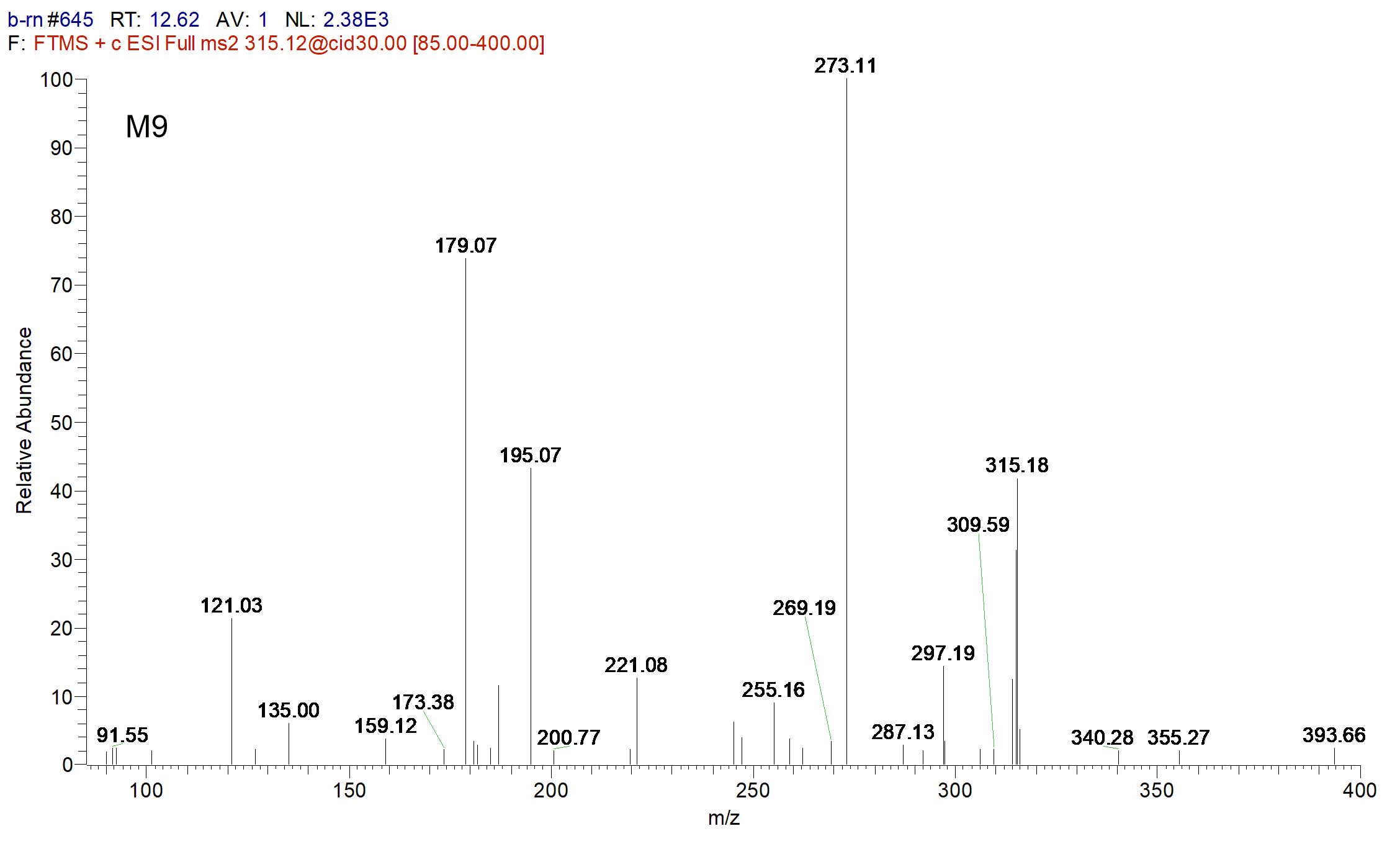


**Supplementary Figure 2.5** MS^2^ spectrum of M9.
