# Supplementary figures and images for: Simulated Microgravity Altered the Metabolism of Loureirin B and the Expression of Major Cytochrome P450 in Liver of Rats
Source: Front Pharmacol. 2018 Oct 12;9:1130. doi: 10.3389/fphar.2018.01130 (PMC6194197; doi:10.3389/fphar.2018.01130)

CYP1A2

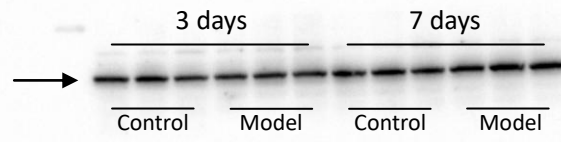

CYP1A2

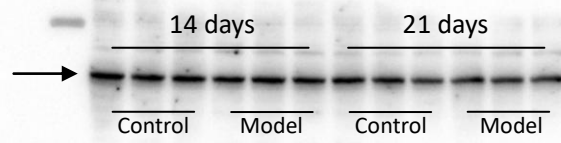

CYP2C11

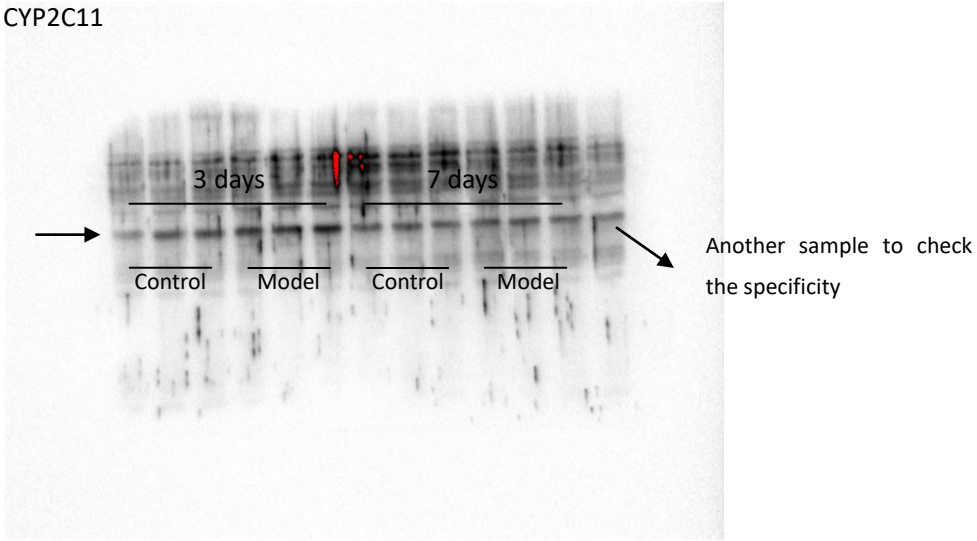

CYP2C11

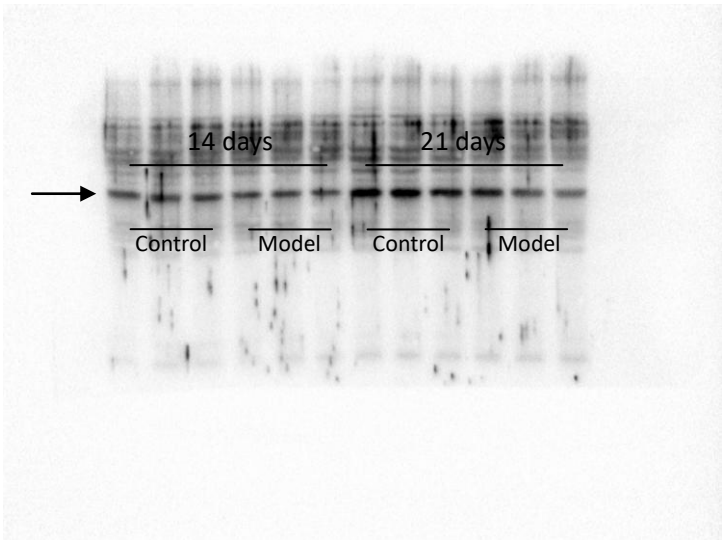

CYP2D1

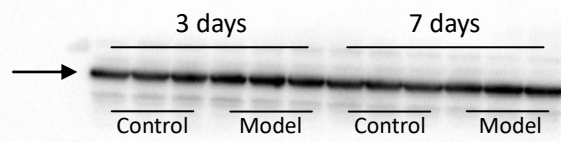

CYP2D1

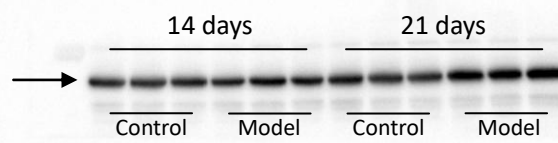

CYP2E1

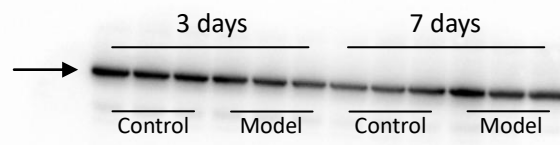

CYP2E1

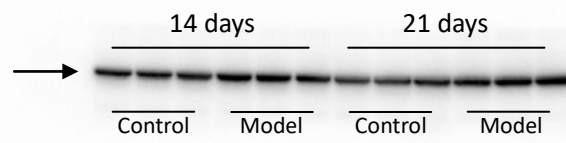

CYP3A2

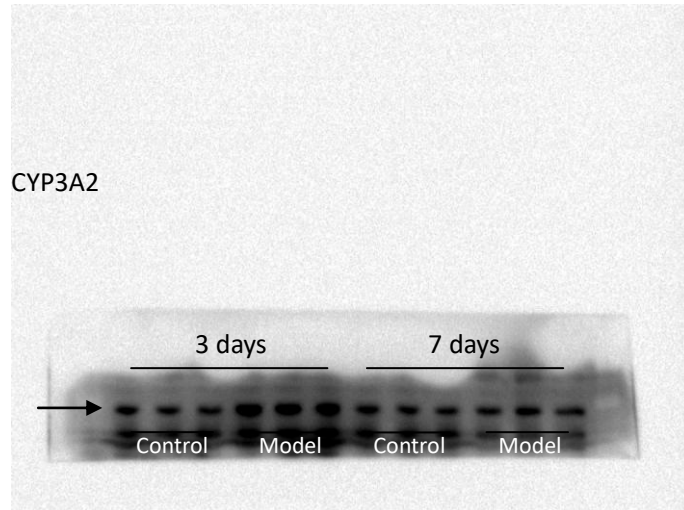

CYP3A2

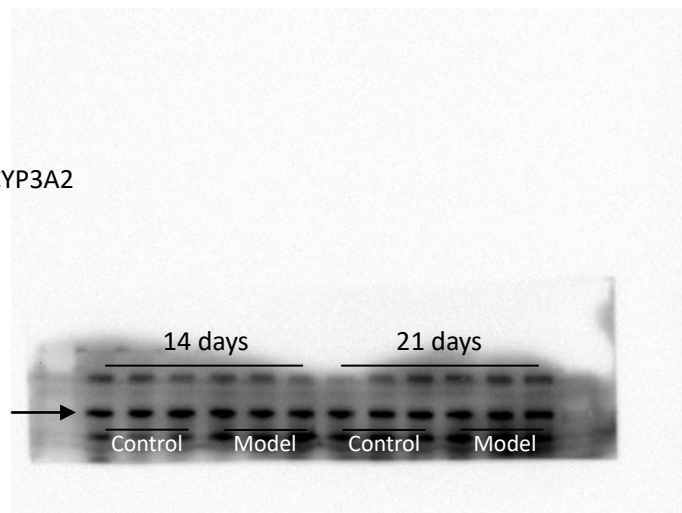

GAPDH

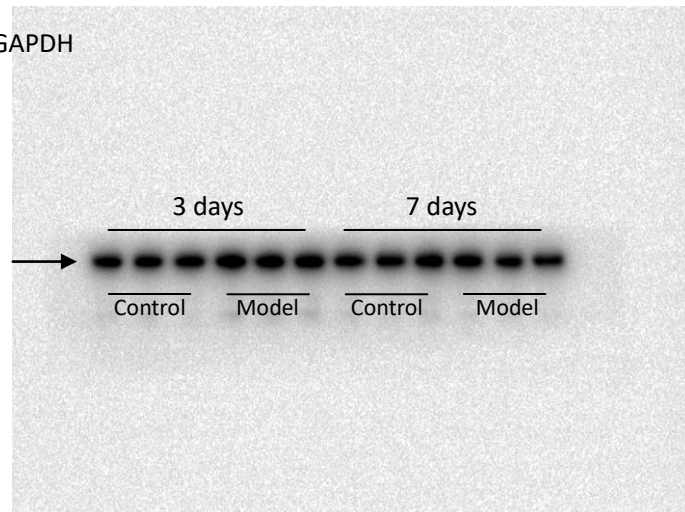

GAPDH

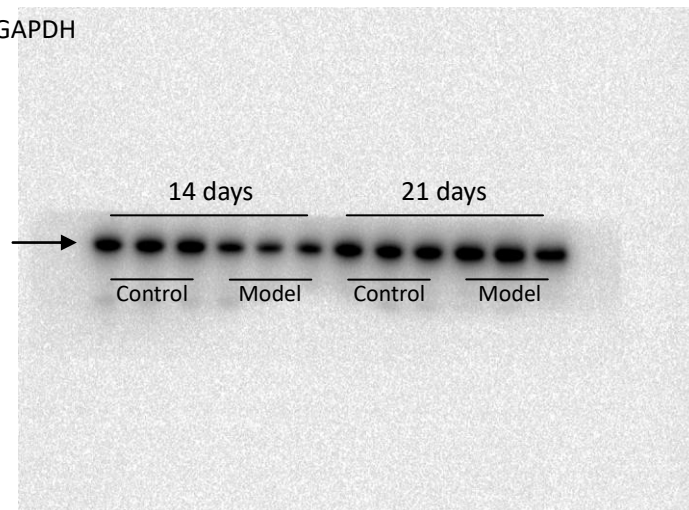

Supplement: DATA SHEET S1 — Original Western blot images. [file Data_Sheet_1.PDF]
